# Self-Raman 1176 nm Laser Generation from Nd:YVO_4_ Crystal by Resonator Cavity Coating

**DOI:** 10.3390/ma16041497

**Published:** 2023-02-10

**Authors:** Fangzheng Qin, Kai Guo, Shihui Ma, Han Zhu, Yixin Lin, Xinkang Dong, Zhenyu Jie, Yonghao Zhu, Yawu Xin, Yongchao Peng, Shifu Xiong, Zhanggui Hu

**Affiliations:** 1 Tianjin Key Laboratory of Functional Crystal Materials, Institute of Functional Crystals, Tianjin University of Technology, Tianjin 300384, China; 2 Optoelectric Component Research and Development Center, Beijing Institute of Control Engineering, Beijing 100094, China

**Keywords:** thin films, self-frequency doubling crystal, ion-assisted deposition, optical monitoring

## Abstract

Crystal coating is an important process in laser crystal applications. According to the crystal characteristics of neodymium-doped yttrium vanadate (Nd:YVO_4_), its intrinsic parameters, and optical film design theory, Ta_2_O_5_ and SiO_2_ were selected separately as high and low refractive index materials. The optical properties and surface roughness of the films were characterized by OptiLayer and Zygo interferometers, and the effects of ion source bias on refractive index and surface roughness were investigated so that the optimal ion source parameters were determined. Optical monitoring and quartz crystal control were combined to accurately control the thickness of each film layer and to reduce the monitoring error of film thickness. The prepared crystal device was successfully applied to the 1176 nm laser output system.

## 1. Introduction

All-solid-state lasers have the characteristics of high efficiency, long life, and small volume. Compared with other types of lasers, it is easier to obtain high-power and high-quality lasers. It has been widely used in coherent optical detection, advanced manufacturing, environmental detection, and optical communication [1,2,3]. The growth and development of neodymium-doped yttrium vanadate (Nd:YVO_4_) can be traced back to 1966 and was the first vanadate used in lasers [4]. Because of its good matching between the absorption line spectrum and the emission spectrum of common LD-pumped light sources, it is often incorporated in the all-solid-state laser system, and its Raman effect is used to obtain fundamental frequency light. It is an ideal gain medium for medium-and small-power lasers [5,6].

Optical thin films play an extremely important role in the research of Nd:YVO_4_ to obtain new wavelengths. They are often used to improve the spectral characteristics of optical systems and are indispensable elements in laser systems. By coating the corresponding optical film on the surface of Nd:YVO_4_ crystal, the self-Raman properties of the crystal can be more effectively coupled and matched, so that the output of a new wavelength Raman laser is possible [7].

In 2020, Shilova et al. used Nd:YVO_4_, lithium triborate (LiB_3_O_5_, LBO) and Ba(NO_3_)_2_ Raman crystals in combination with a laser system, and a 563 nm laser by coating a filter/reflection film on the LBO crystal was successfully excited [8]. In 2021, Wu et al. achieved a dual-wavelength switchable output of 1064/1342 nm by adding two sets of photoelectric conversion Q-switched RTP (RbTiOPO_4_) based on an LD + Nd:YVO_4_ all-solid-state laser system [9]. Chang et al. fabricated a pulse width Q-switched device using graphene and achieved a stable Q-switched output of 1064.1 nm laser based on Nd:YVO_4_ all-solid-state laser [10]. In 2022, Zhu et al. used YVO_4_ composite crystal as a gain medium to improve the problem of poor thermal diffusion of Nd:YVO_4_, and successfully obtained a 589 nm laser [11]. Wu et al. used YLF (yttrium lithium fluoride, LiYF_4_) and YVO_4_ as gain medium, and successfully obtained a 2052 nm laser with a maximum output power of 3.3 W using a 792 nm pump light source [12].

Based on the above research work, it can be found that Nd:YVO_4_ crystals are a very promising material for developing Raman lasers with practical value. In this work, based on the self-Raman effect of Nd:YVO_4_ and optical thin film design theory, Ta_2_O_5_ and SiO_2_ are used as high and low refractive index materials, respectively, for resonant cavity coating, and the 1176 nm Raman laser is successfully output. Meanwhile, the effect of ion source bias on refractive index and surface roughness is investigated to achieve the best output power for the self-Raman effect. This work has broadened the application spectrum of yttrium vanadate and lays a solid foundation for the acquisition of new wavelengths of fundamental frequency light for all-solid-state laser systems.

## 2. Thin-Film Material

**Figure 1:**
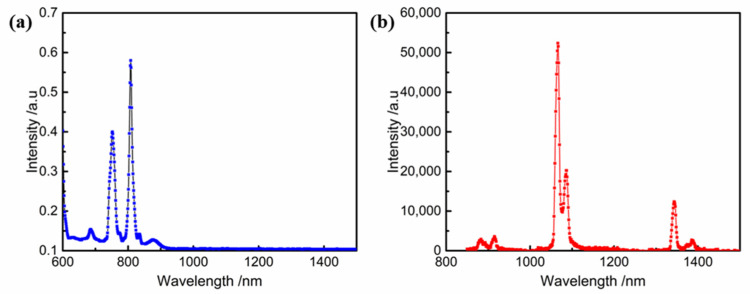
(**a**) Absorption and (**b**) emission spectra of Nd:YVO_4_ crystal at room temperature.

Nd:YVO_4_ crystal is a kind of laser crystal with excellent performance, which belongs to the tetragonal system and positive uniaxial crystal. The unit cell parameters of the crystal are a = b = 7.1193 nm, c = 6.2892 nm. Its Mohs hardness is about 4~5. It has the characteristics of high absorption coefficients, a large stimulated emission cross-section, high slope efficiency, high photodamage resistance, and deliquescence proof. It has a refractive index of 2.021(@1064 nm) and a high optical transmittance in the range of 400~5000 nm [13]. Doping different elements and controlling the doping concentration can determine the Raman conversion pattern of YVO_4_, where single doping or co-doping of Yb^3+^, Er^3+^, Ce^3+^, and Ho^3+^ ions can make YVO_4_ achieve up-conversion luminescence, while Eu^3+^ and Nd^3+^ ions can achieve down-conversion luminescence [14]. The absorption and emission spectra of Nd:YVO_4_ crystal samples at room temperature used in this work are shown in Figure 1.

**Figure 2:**
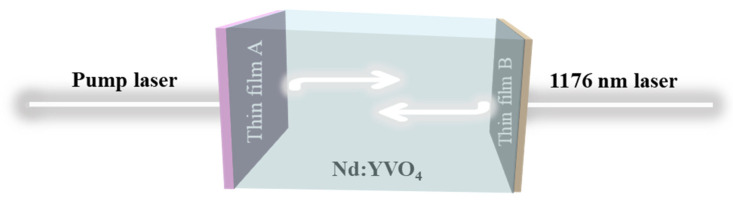
Working diagram of Nd:YVO_4_ crystal device generating self-Raman 1176 nm laser.

As can be seen in Figure 1a,b, the absorption peak of Nd:YVO_4_ is around 810 nm, and the emission peak is around 1064 and 1342 nm, indicating that Nd:YVO_4_ can achieve down-conversion luminescence. Because Nd:YVO_4_ crystal has the characteristics of a large emission cross-sectional area and high absorption coefficient, the quality of the film surface deposited on the crystal surface will directly affect the final laser quality and service life. According to the requirements of the all-solid-state laser system, the working diagram of Nd:YVO_4_ crystal device is shown in Figure 2. The specific technical parameters of the optical film on the crystal end face are shown in Table 1.

Accord requirements to the technical parameters shown in Table 1, the selected coating materials meet the requirements of the transparent region in the near-infrared band, and the factors such as refractive index, mechanical properties, film stress matching and film stability are considered [15]. In the near-infrared band, the common high refractive index materials are TiO_2_, ZrO_2_, HfO_2_, and Ta_2_O_5_. Among them, TiO_2_ has the highest refractive index in this band, but multivalent oxides of titanium are easy to appear in the coating process, and the optical constants of the films are greatly affected by the coating environment [16]. ZrO_2_ has the problems of unstable deposition rate and low damage threshold. HfO_2_ has the characteristics of low laser absorption and a high damage threshold [17]. However, it tends to produce phase changes during evaporation and is accompanied by sputtering phenomena, leading to the formation of nodular defects on the film surface. Ta_2_O_5_ has the characteristics of a high damage threshold, low absorption, and stable physical and chemical properties. Ta_2_O_5_ film has a more stable saturated Ta-O bond structure, and a longer structural delay time than HfO_2_ film under 1064 nm laser radiation; that is, they are less prone to phase transition under laser radiation [18]. Taken together, Ta_2_O_5_ is chosen as the high refractive index material.

MgF_2_ and SiO_2_ are commonly used low refractive index materials. Among them, the MgF_2_ film is hard and durable, but it is prone to splash during evaporation. When the cumulative thickness exceeds 1.4 μm, the larger tensile stress causes the film to crack [19]. SiO_2_ film has the characteristics of stable performance, a high laser damage threshold, and low absorption loss [20,21]. Both SiO_2_ and Ta_2_O_5_ are oxides, and if they are used as low and high refractive index materials, respectively, the negative effects caused by oxygen charging during the preparation of Ta_2_O_5_ films can be effectively reduced or even avoided. Therefore, SiO_2_ is selected as the low refractive index coating material.

## 3. Theoretical Design of a Resonant Cavity Film System

For a multi-period film A,B^S (where *A*, *B* represent the film layers of two different materials, *S* is the corresponding film stack repetition period), the optical admittance of the medium around a single period is η, and its characteristic matrix is

(1) $$M=m_{A}\cdot m_{B}=\left[\begin{array}{cc} \cos\delta_{A} & \frac{i}{\eta }\sin\delta_{A}\\ i\eta \sin\delta_{A} & \cos\delta_{A} \end{array}\right]\cdot \left[\begin{array}{cc} \cos\delta_{B} & \frac{i}{\eta }\sin\delta_{B}\\ i\eta \sin\delta_{B} & \cos\delta_{B} \end{array}\right]=\left[\begin{array}{cc} m_{11} & m_{12}\\ m_{21} & m_{22} \end{array}\right]$$

where $\;m_{A}$,$m_{B}$ is the characteristic matrix of the film,$\;\delta_{A}$, $\delta_{B}\;$ is the phase of the film,$\;m_{11}$,$\;m_{12}$, $m_{21}$ and $\;m_{22}$ are its matrix elements. The transmission coefficient for this period is

(2) $$t=\frac{2\eta }{\eta \left\{\left(m_{11}+m_{22}\right)+\left[\eta m_{12}+\left(m_{21}/\eta \right)\right]\right\}}$$

Let $t=\left|t\right|\exp\left(i\tau \right)(\tau$ be the phase change of the transmitted light,  τ≈∑δ),$\;e^{i\tau }=\cos\tau +i\sin\tau$ organized by substitution (2), then

(3) $$\frac{1}{2}\left\{\left(m_{11}+m_{22}\right)+\left[\eta m_{12}+\left(m_{21}/\eta \right)\right]\right\}=\frac{\cos\tau -i\sin\tau }{\left|t\right|}$$

When the dielectric film has no absorption or the absorption can be ignored, the $m_{11}$ and $m_{22}$ are real numbers, the $m_{12}$ and $m_{21}$ are pure imaginary numbers. Let the real part be equal, so

(4) $$\frac{1}{2}\left(m_{11}+m_{22}\right)=\frac{\cos\tau }{\left|t\right|}$$

It can be seen that when ∑δ=mπ, then  cosτ=±1. If $\left|t\right|<1$, $\;\frac{1}{2}\left|m_{11}+m_{22}\right|>1$, a reflection band is formed at the corresponding reference wavelength; when $\left|t\right|=1$,$\frac{1}{2}\left|m_{11}+m_{22}\right|=1$, the reflection band of the corresponding reference wavelength will be suppressed to form a transparent band.

For the technical requirements of laser crystal multi-band filtering, it is necessary to introduce a plurality of multi-period film systems with different reference wavelengths, and according to this theory, the transmittance of the band has a frequency doubling relationship (such as  λ/2, λ/3  etc.) with the reference wavelength regulated to achieve technical indicators.

### 3.1. Input Surface Design

The input surface requires high transmission at 808 nm, 880 nm, 916 nm, and 1342 nm, and high reflection at 1064 nm and 1176 nm. For this index, a short-wave pass can be used as the basic film system, set as Sub|q (0.5 LH 0.5 H)^^S^|Air, where q is the corresponding film stack coefficient, S is the corresponding film stack repetition period, Sub is Nd:YVO_4_ crystal, H is Ta_2_O_5_ with optical thickness of 1/4 wavelength, and L is SiO_2_ with optical thickness of 1/4 wavelength. According to the reflection bandwidth of the film system and the reference wavelength, the film stack coefficient and the number of cycles of the basic film system are determined. When S ≥ 20, the high-reflection region can obtain a higher cutoff. The initial film system of the parameter is Sub|(0.5 LH 0.5 H)^^20^|Air, and the reference wavelength λ_0_ is 1100 nm.

**Figure 3:**
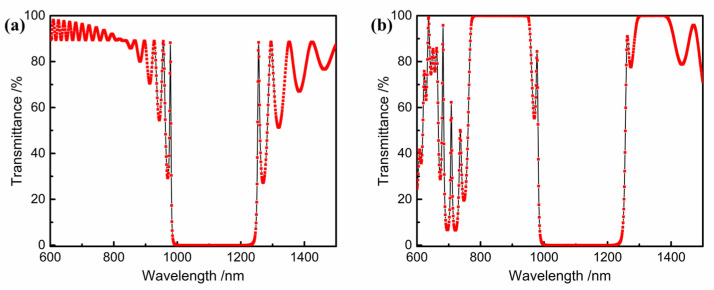
(**a**) The initial film system transmittance spectral curve and (**b**) the theoretically calculated transmittance spectral curve of multilayer optical coatings for the input surface.

According to the technical parameters, the optimization continuous target is set in OptiLayer software, and the input surface theory design spectral curve is presented in Figure 3a. On the basis of the main structure of the regular film system, an irregular film layer is introduced to compress the band-pass interval ripple and improve the transmittance. The final film system is Sub|1.3666 H 1.6566 L 0.2396 H 1.3894 L···1.6920 H 0.6675 L 2.7674 H 0.6257 L|Air, a total of 46 layers. The physical thickness is about 8.1 μm, and the theoretical transmittance spectrum curve is shown in Figure 3b. The average transmittance of the 790~940 nm band is 99.97%, the average reflectivity of the 1000~1210 nm band is 99.98%, and the average transmittance of the 1310~1370 nm band is 99.97%, which meets the design requirements.

### 3.2. Output Surface Design

**Figure 4:**
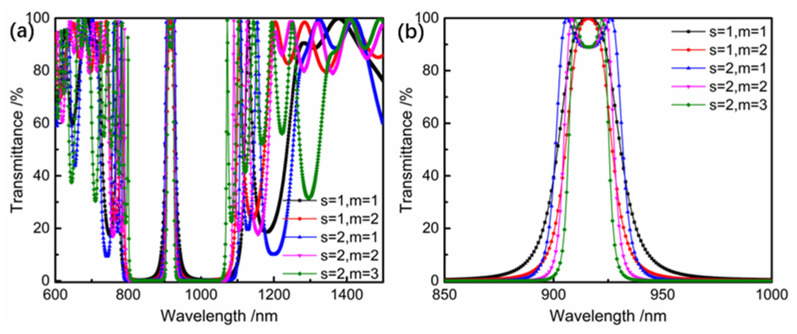
Transmittance spectral curve of the initial film system on the output surface (**a**) 600~1500nm, (**b**) 850~1000 nm.

According to the index parameters, 910~920 nm, and 1300~1380 nm are the high transmission bands, 780~890 nm and 1000~1120 nm are the high reflection bands, and the transmission (T) of the 1170~1210 nm band is 1%. Due to the high transmittance at a 916 nm wavelength and the cut-off of both sides, the Fabry-Perot interference method is selected to achieve a higher cut-off with fewer layers [22]. With the increase in the interference order, the full width at half the maximum of the band-pass becomes narrower, and with the increase in the number of repetition cycles, the band-pass width becomes wider. Therefore, the band-pass width requirement can be achieved by adjusting the interference order and the number of repetition cycles. The F-P base film system is Sub|(HLHLH 2 mL HLHLH L)^^s^ HLHLH 2 mL HLHLH|Air, where m is the interference level, s is the number of repetition cycles of the reflective film and the spacer layer, and the highest interference level is usually used to the third level. After simulation, when s = 1, the cutoff of the 780~890 nm band is lower than the required value. When s ≥ 2, the cutoff band on both sides of the narrow band basically meets the requirements. At this time, the number of film layers increases with the increase in the s value. Therefore, s = 2 is taken in this work. When m = 1, s = 2, the initial film parameters and indicators are basically matched; when m = 2, s = 2, the transmission band at 1010~1100 nm is too narrow to meet the parameter requirements. When m = 3, s = 2, all transmission bands are too narrow to meet the parameter requirements, as shown in Figure 4. Therefore, the basic membrane system with m = 1 and s = 2 is determined as the initial membrane system.

**Figure 5:**
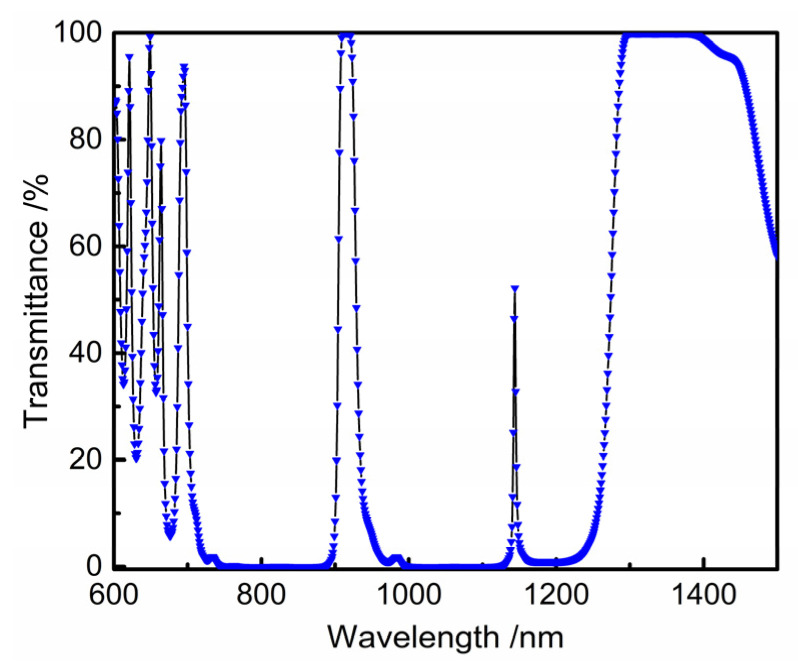
Theoretically calculated transmittance spectral curve of multilayer optical coatings for the output surface.

Based on the Fabry-Perot film system, a short-wave membrane system is superimposed to meet the 1176 nm part of the transmission requirements. The basic film system is Sub|HLHLH2LHLHLH L HLHLH2LHLHLH L HLHLH2LHLHLH 1.18 (0.5 LH 0.5 L)^^10^|Air. The optimized film system is Sub|1.0160 H 0.8710 L 0.7802 H 0.8648 L···1.1597 H 1.0922 L 0.9896 H 2.4209 L|Air, with 56 layers and a physical thickness of about 7.89 μm. The transmittance spectrum curve is shown in Figure 5. The average reflectance of the 780~890 nm band is 99.96%; the average transmittance of the 910~920 nm band is 99.97%; the average reflectance of the 1000~1120 nm band is 99.96%; the average transmittance of the 1170~1210 nm band is 1.00%; and the average transmittance of the 1300~1380 nm band is 99.99%, which meets the design requirements.

## 4. Thin Film Preparation and Analysis

The experiment was carried out on a Leybold SYRUSpro1110 vacuum coating machine equipped with a double ‘e’ electron gun, an optical film thickness monitoring system, a six-probe crystal control system, an auxiliary ion source, and a double resistance evaporation source.

The transparent surface of Nd:YVO_4_ crystal was cleaned with a mixture of ethanol and ether (volume ratio of 1:3). After the vacuum chamber was cleaned and the filler was completed, the YVO_4_ crystal was placed on the fixture in the vacuum chamber. When the vacuum degree reached 8 × 10^−2^ Pa, turned on the baking. When the temperature reached 180 °C, it needed to hold for 20 min. The ion source was opened to pre-clean crystal for 180 s, and began coating.

### 4.1. Optimization of the Electron Beam Premelting Path

**Figure 6:**
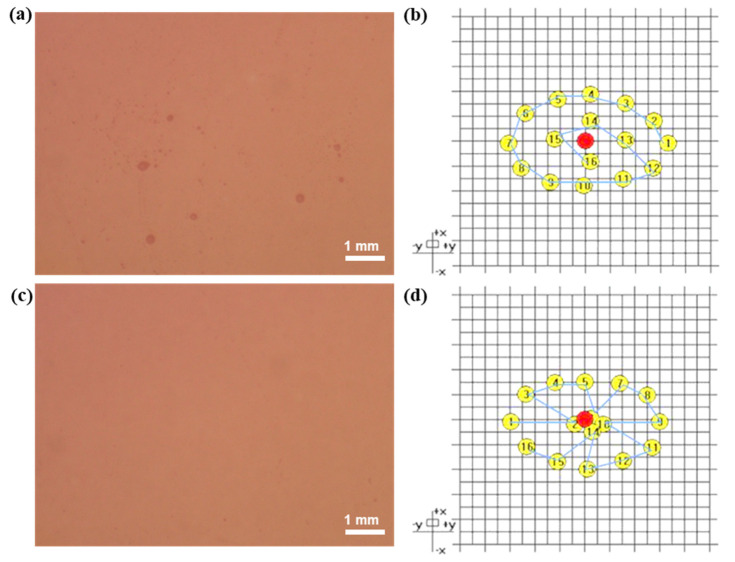
(**a**) Micrograph of the film surface before optimization. (**b**) Original diagram of the electron beam melting path. (**c**) Micrograph of the optimized film surface. (**d**) Optimized diagram of the electron beam melting path, the obscured numbers are 2, 6, 10 and 14 in clockwise order. The numbers 1–16 indicate the trajectory of the electron beam.

The surface morphology of the single-layer Ta_2_O_5_ film was observed by an OLYMPUS DP74 fluorescence microscope, and it was found that there were spray points on the surface of the film, as shown in Figure 6a. Combined with the defect characteristics of the coating and the analysis of the deposition process, it was found that the pre-melting degassing of the Ta_2_O_5_ material was not sufficient. The uneven heating of the film material during vapor deposition leads to localized spattering, thus forming this spray point defect on the film surface. The operation path of the electron beam melting material is analyzed, as shown in Figure 6b. It is found that the electron beam route does not effectively cover the entire crucible area. Instead, it is mainly concentrated on the edge area. The material that is not covered by the path at the center of the dense accumulation will have insufficient pre-melting.

In order to solve this problem, the operation path of the electron beam is re-optimized. After several optimized experiments, the final pre-melting scheme is shown in Figure 6d. This scheme can ensure that the effective operation trajectory of the electron beam covers the entire crucible material surface and that the material can be fully pre-melted. According to the optimized pre-melting process, the film samples were re-prepared. As shown in Figure 6c, the defects on the film surface are significantly reduced, and the surface quality is greatly improved.

### 4.2. Optimization of Film Surface Roughness

Ion beam-assisted deposition is a common technique for preparing thin films with low absorption and high quality [23,24,25,26]. Combined with thermal evaporation, it can improve the optical and mechanical properties of thin films and obtain dense, uniform, and stable films. For ion beam-assisted deposition, ion energy has a significant effect on the structure of the film, and the ion source bias voltage is the direct manifestation of ion energy. Therefore, we investigate the relationship between the refractive index and surface morphology of the films and the bias voltage of the ion source, and the quality of Ta_2_O_5_ films is optimized by changing the bias voltage of the ion source.

The ion source bias voltage of the single-layer Ta_2_O_5_ film was set to 90, 100, 110, 120, 130, and 140 V, respectively, and the other parameters were kept consistent. The substrate temperature was 180 °C, and the deposition rate was 0.3 nm/s. The film thickness was controlled by a quartz crystal oscillator. The Ar flow rate was 12 mL/min, and the O_2_ flow rate was 30 mL/min.

**Figure 7:**
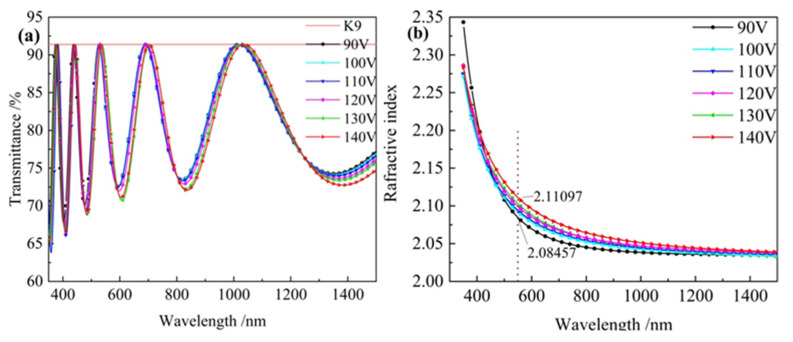
Transmittance and refractive index curve of single-layer Ta_2_O_5_ film at different ion source bias. (**a**) Transmittance; (**b**) Refractive index.

The transmittance of the monolayer Ta_2_O_5_ films was tested at different ion source bias pressures, as shown in Figure 7a. It can be observed that the maximum transmittance at the 550 nm band at different bias pressures is close to that of K9 glass, which indicates that the refractive indices of the prepared films are uniformly distributed, obeying the dispersion law. Simultaneously, with an increase in bias voltage, the transmittance spectrum curve moves toward the long wave direction. The refractive index of Ta_2_O_5_ films under different bias voltages was calculated using MCalc software, as shown in Figure 7b. The refractive index increases with an increase in bias voltage.

The variation in the bias voltage of the ion source affects the denseness of the film, which further leads to the variation in the refractive index of the film. The denseness of a film is expressed in the optical field by the aggregation density. It has been reported that the film layers of optical films almost always have a significant columnar structure, which runs across the film and is perpendicular to the interface. The aggregation density is defined as the ratio of the volume of the solid column to the volume of the film, with values typically in the range of 0.75 to 1.0. The refractive index of the film is a function of the aggregation density, and the simplest linear approximation is given by Equation (5).

(5) $$n_{f}=pn_{s}+\left(1-p\right)n_{v}$$

where $n_{f}$ is the refractive index of the material in the solid state, *p* is the aggregation density, $n_{s}$ is the refractive index of the material in the solid state, and for Ta_2_O_5_, $n_{s}\;$= 2.14 at 550 nm, $n_{v}$ is the refractive index of air, $n_{v}$= 1.

It can be seen that the aggregation density of the film at different bias pressures follows the relationship of Formula (6).

(6) $$p=\frac{n_{f}-n_{v}}{n_{s}+n_{v}}$$

The aggregation density is calculated according to Formula (6), and the results are shown in Table 2.

**Figure 8:**
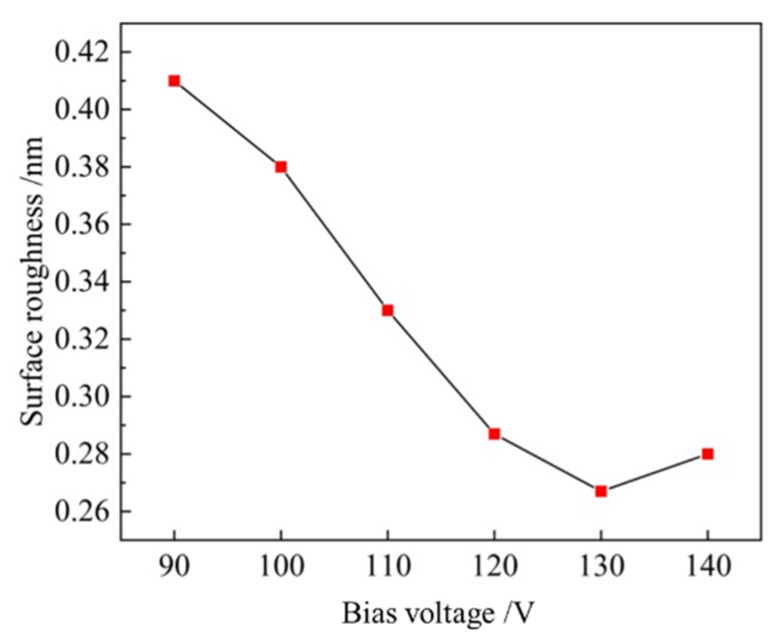
Surface roughness of Ta_2_O_5_ films under different ion source bias.

Surface roughness of Ta_2_O_5_ thin films prepared under different bias voltages was characterized by Zygo white light interferometer. The results are shown in Figure S1 in the supporting information. When the bias voltage increases from 90 V to 130 V, the root mean square roughness (Sa) of the single-layer Ta_2_O_5_ film decreases from 0.410 nm to 0.267 nm. When the bias voltage continues to increase to 140 V, the surface roughness tends to increase, which may be due to the etching of the film surface by the ion source. Therefore, an ion source bias voltage of 130 V is selected for this work (Figure 8). The ion source parameters for Ta_2_O_5_ and SiO_2_ film deposition are shown in Table 3, where Ar flow 1 is the argon supply inside the ion source, while Ar flow 2 is the rate of argon supply inside the vacuum chamber (around the ion source).

### 4.3. Film Thickness Monitoring Scheme

At present, film thickness control is mainly divided into quartz crystal monitoring and optical monitoring [27]. Quartz crystal monitoring measures the physical thickness of the film by monitoring the changes in the vibration frequency of the AT-cut quartz crystal [28]. The signal changes linearly, and it is easy to monitor the deposition rate. With the increase of film thickness, the vibration frequency of quartz crystal decreases, and the detection sensitivity decreases accordingly, resulting in subtle errors. These errors have little effect on the total thickness of the thinner film. However, the cumulative thickness error will have a greater impact on the final result for the thicker film. The optical monitoring method is to invert the optical thickness of the thin film by using the intensity change or polarization change of transmitted light or reflected light caused by thin film interference [29,30]. The optical thickness of the film and the compensation mechanism for the film thickness error can be provided by the optical control method, but the signal changes sinusoidally, and it is difficult to monitor the deposition rate.

In the actual coating process, the preparation of each layer differs from the theoretical design due to the influence of vacuum, baking temperature, rate, and other factors. Assuming that the target transmittance of the optical film to be plated is $T^{th}\left(n,d\right)$ (*n* is the refractive index of the film material of the layer, d is the physical thickness of the film of the layer), and the transmittance actually measured during the coating process is $T^{ex}\left(n,d\right)$, the evaluation function for film thickness monitoring can be expressed as

(7) $$F\left(d\right)=\left|T^{th}\left(n,d\right)-T^{ex}\left(n,d\right)\right|$$

For the i-th layer film, the evaluation function is

(8) $$F_{i}\left(d\right)=\left|T_{i}{}^{th}\left(n,d\right)-T_{i}{}^{ex}\left(n,d\right)\right|$$

During the film deposition process, the spectrometer will continuously and quickly obtain the measured film transmittance spectrum $T_{i}{}^{th}\left(n,d\right)$ that changes with time, and then transmit the data to the computer to calculate the evaluation function at this time. As the film deposition thickness increases, the measured transmittance value gradually approaches the theoretically calculated value, and the evaluation function value decreases. When the measured transmittance spectrum reaches the theoretical calculation value, the evaluation function obtains the minimum value, and it is judged that this time is the stop time point, and the deposition of the film ends. Next, the computer re-fits the next layer of the film based on the transmittance value at this point. The next layer parameters determine the termination transmittance value for the next layer of film, and then proceed to the next layer of film. The above procedure is repeated until all layers are deposited.

In this work, a combination of the optical monitoring method and the quartz crystal monitoring method is used. The optical control monitors the film thickness, and the crystal control monitors the deposition rate. The design parameters are imported into MCalc software, and the number of photocontrols, monitoring wavelength, and other parameters are optimized to obtain the optimal monitoring scheme. The number of optical controllers should be as small as possible while meeting the monitoring requirements to reduce the cumulative error caused by switching optical controllers during the film deposition process. Monitoring wavelength is as much as possible the same or close to reduce the system error of grating adjustment.

**Figure 9:**
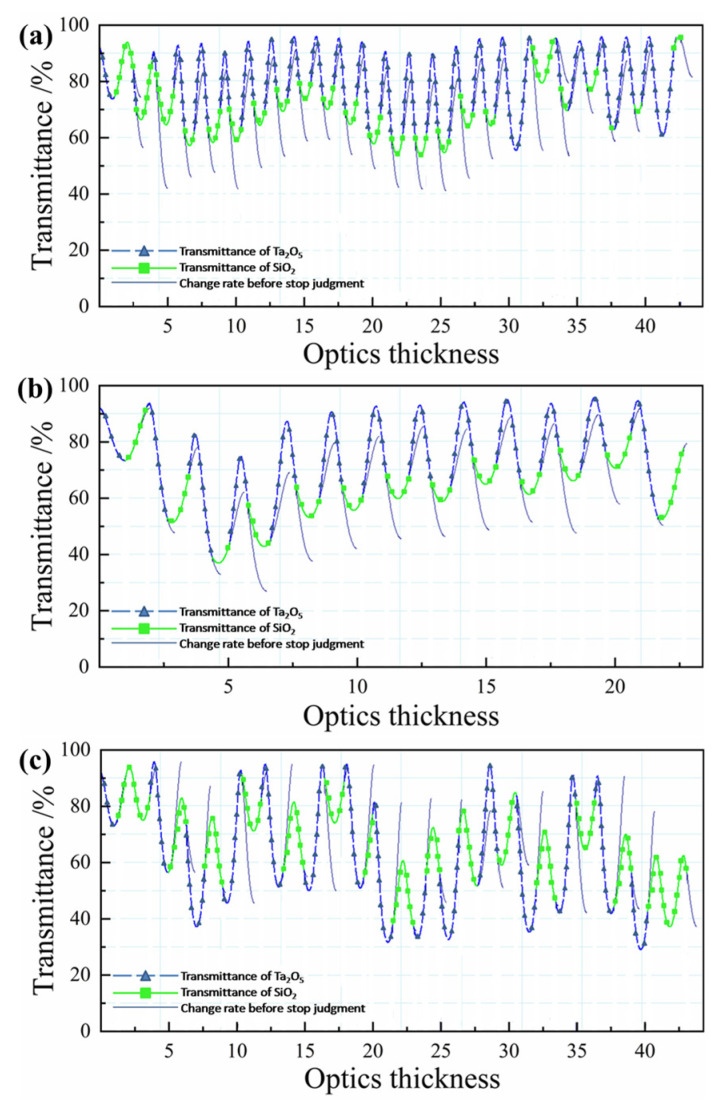
(**a**) Optical monitoring design of the input surface film system. Optical monitoring design of output surface film system (**b**) 1^#^ and (**c**) 2^#^.

Combined with the sensitivity analysis of the input surface film layer through simulation analysis, when the monitoring wavelength is 1300 nm, the error of each film layer can be controlled within 1%. The optical signal changing with thickness is shown in Figure 9a. It can be seen that the monitoring scheme is reasonable and feasible.

Similarly, the monitoring scheme is analyzed based on the sensitivity distribution of the output mask layer. It is found that when the number of optical controllers is less than 2, the monitoring scheme with a 1% error of each layer cannot be found. Therefore, the number of optical control chips in the monitoring scheme is determined to be 2, and the 1st to 26th layers and the 27th to 56th layers are monitored by one optical control chip, respectively. The monitoring wavelength of 1^#^ optical control chip is 925 nm, and the monitoring wavelength of 2^#^ optical control chip is 770 nm. The variation in the optical signal with thickness is shown in Figure 9b,c).

## 5. 1176 nm Laser Output

**Figure 10:**
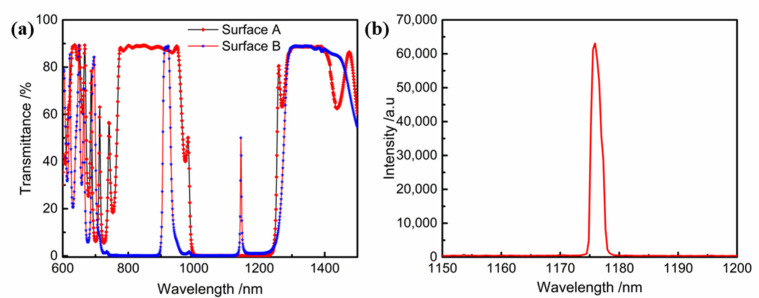
(**a**) Measured transmittance spectral curve of Nd:YVO_4_ crystal with single-sided coating. (**b**) 1176 nm laser output pattern obtained under 808 nm laser excitation.

The sample was detected by an Agilent Cary7000 UV-visible spectrophotometer. The sample was a single-sided coated YVO_4_ crystal (when the second surface was not coated, the substrate/air interface reflectance was 10.47% @532 nm, 10.69% @808 nm, 11.42% @1064 nm). The spectral curve is shown in Figure 10a. 

The average transmittance of the input plane in the 780~950 and 1300~1380 nm bands are 88.1% and 88.6%, respectively, and the average reflectance in the 1000~1230 nm band is 99.5%. The average transmittance of the output plane in the 910~920 and 1170~1210 nm bands are 87.9% and 88.6%, respectively. The average reflectance in the 780~890 and 1000~1120 nm bands are 99.9% and 99.9%, respectively, and the average transmittance in the 1170~1210 nm band is 1.0%. The spectral test results meet the indicator requirements. 

In this experiment, the pump source was a fiber-coupled laser diode with a central wavelength of 808 nm. The diameter of the fiber was 200 μm with a numerical aperture of 0.22. A 1:1 focusing system was applied to focus the pump light into the gain medium. The gain medium was an a-cut 4 at.% doped Nd:YVO_4_ crystal with dimensions of 3 × 3 × 8 mm^3^. The coated YVO_4_ crystal device was placed in the optical system, and an 1176 nm laser output was successfully obtained by 808 nm laser excitation. The wavelength test is shown in Figure 10b.

Because stimulated Raman scattering belongs to the third-order nonlinear optical effect, pulse lasers with peak power are generally selected as the pump light source, so the Q-switching device needs to be added in the previous design, but this will also increase the cavity length, and cavity loss. In our micro-cavity design, we will only use a single crystal to realize the self-Raman laser output. By directly coating the film system on the crystal and removing the Q-switching elements. A shorter cavity length is obtained, but the conversion efficiency will be decreased owing to continuous optical pumping. Finally, this design conforms to the development trend of integration and miniaturization design and provides another technical solution for the self-Raman laser.

## 6. Conclusions

Based on the basic theory of thin film, the number of cycles is discussed and analyzed, the basic film system is determined, and the optimization design is carried out by further combining with OptiLayer software. The melting process of Ta_2_O_5_ material was studied, and the melting path was optimized to reduce the spray point on the film surface. The influence of the ion source-assisted process on the refractive index and surface roughness of the film was analyzed. The ion source parameters were optimized, and the surface roughness was reduced. Using MCalc software combined with the sensitivity of the film, the optical control scheme was designed, and the prepared Nd:YVO_4_ crystal device achieved an 1176 nm laser output. The quality of the film affects the conversion efficiency of the frequency-doubling laser. By annealing and optimizing the deposition rate of the film, further reducing the surface roughness of the film and improving the transmittance of the transmission band will be the focus of future research.

## Figures and Tables

**Table 1:** Technical parameters of resonant cavity film based on Nd:YVO_4_ crystal for self-Raman 1176 nm laser generation.

Parameter	Surface A	Surface B
Substrate	Nd:YVO_4_	
Angle of incidence/(°)	0	
High transmission band/nm	808 & 880 & 916 & 1342	808 & 880 & 1064
Transmittance/%	≥98	
High cutoff band/nm	1064 & 1176	916 & 1342
Reflectivity/%	≥99	
Partially transmissible band/nm	-	1176
Transmittance/%	-	1 ± 0.2

**Table 2:** Refractive index and packing density of Ta_2_O_5_ films under different ion source bias volt ages.

Bias Voltage/V	Refractive Index@550 nm	Packing Density
90	2.084	0.950
100	2.091	0.957
110	2.094	0.959
120	2.099	0.964
130	2.102	0.966
140	2.110	0.973

**Table 3:** Process parameters of the ion source for depositing Ta_2_O_5_ and SiO_2_.

Material	Bias Voltage /V	Coil Current/A	Discharge Voltage/V	Discharge Current/mA	Ar Flow 1 / (mL·min^−1^)	Ar Flow 2 / (mL·min^−1^)
Ta_2_O_5_	130	1.45	83	50	5.3	6.5
SiO_2_	160	1.80	130	55	5.0	7.0

## Data Availability

The data in this research are available from the corresponding author upon reasonable request.

## Supplementary Materials

The following supporting information can be downloaded at: https://www.mdpi.com/article/10.3390/ma16041497/s1, Figure S1. Surface roughness (Sa) of Ta_2_O_5_ films prepared with Zygo white light interferometer at different bias voltages are (a) 0.410 nm for 90 V, (b) 0.381 nm for 100 V, (c) 0.331 nm for 110 V, (d) 0.287 nm for 120 V, (e) 0.267 nm for 130 V, and (f) 0.280 nm for 140 V.

## Abbreviations

*M*	Characteristic matrix of film
*η*	Optical admittance
*δ*	Phase of the film
*τ*	Phase change of the transmitted light
*λ*	Wavelength of electromagnetic wave
LD	Laser Diode
Ar	Argon gas
O_2_	Oxygen
Sa	Root mean square roughness
