# Supplementary material for: Self-Raman 1176 nm Laser Generation from Nd:YVO4 Crystal by Resonator Cavity Coating
Source: Materials (Basel). 2023 Feb 10;16(4):1497. doi: 10.3390/ma16041497 (PMC9964753; doi:10.3390/ma16041497)
Supplement: Supplementary file 1 [file materials-16-01497-s001.zip › materials-2180180-supplementary.pdf]

Supporting information

Self-Raman 1176 nm Laser Generation from Nd:YVO_4 Crystal by Resonator Cavity Coating

Fangzheng Qin [1], Kai Guo[2], Shihui Ma [1], Han Zhu [1], Yixin Lin [1], Xinkang Dong [1], Zhenyu Jie [1], Yonghao Zhu [1], Yawu Xin [1], Yongchao Peng [1], Shifu Xiong [1,*] and Zhanggui Hu [1]

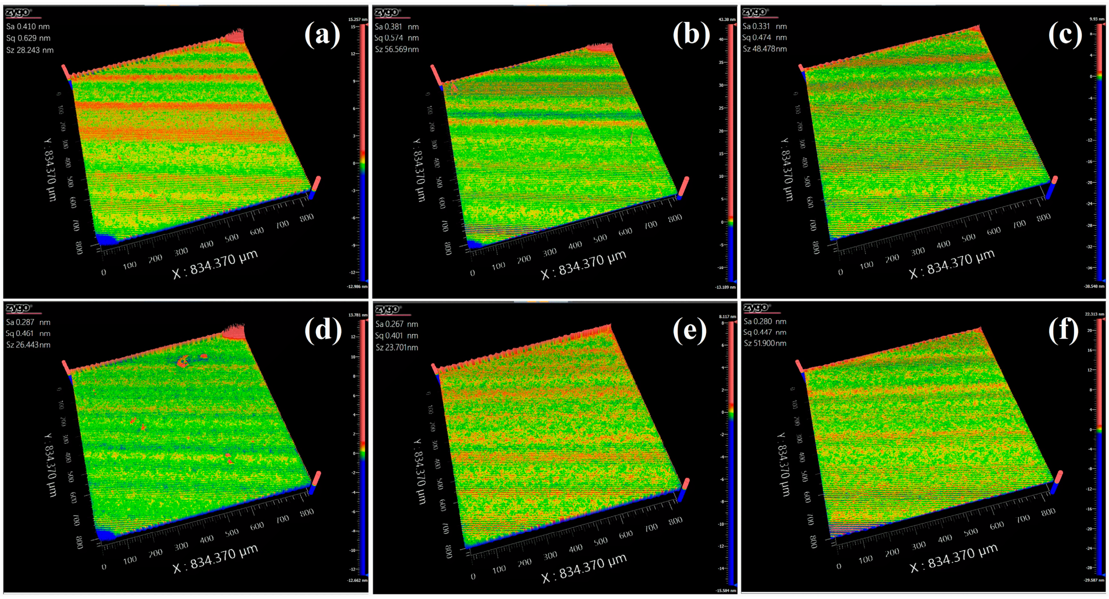

Figure S1. Surface roughness (Sa) of Ta_2O_5 films prepared with Zygo white light interferometer at different bias voltages are (**a**) 0.410 nm for 90 V, (**b**) 0.381 nm for 100 V, (**c**) 0.331 nm for 110 V, (**d**) 0.287 nm for 120 V, (**e**) 0.267 nm for 130 V, and (**f**) 0.280 nm for 140 V.
